# Editorial: Innate Immune Cells and Inflammatory Mediators in Mucosal Pathologies

**DOI:** 10.3389/fimmu.2020.01679

**Published:** 2020-07-28

**Authors:** Leite-de-Moraes Maria, Chieppa Marcello, Vermeulen Mònica

**Affiliations:** ^1^Laboratory of Immunoregulation and Immunopathology, Institut Necker-Enfants Malades (INEM), CNRS UMR8253 and Inserm UMR1151, Paris, France; ^2^National Institute of Gastroenterology “S. de Bellis”, Research Hospital, Castellana Grotte, Italy; ^3^Laboratorio de Células Presentadoras de Antígeno y Respuesta Inflamatoria, IMEX (Instituto de Medicina Experimental)-CONICET, Academia Nacional de Medicina, Buenos Aires, Argentina

**Keywords:** inflammation, innate cells, mucosal pathology, mediators, homeostasis

Cells of the innate immune system play an essential role in early protection against pathogens and tissue damage. They are also in the front line for maintaining the fragile equilibrium between inflammatory responses and control of host body hemostasis. In this special issue you will find a number of original, complementary approaches aiming at a better understanding of how innate cells modulate inflammatory responses. Contributions focus mainly on mediators produced by these cells as well as mechanisms through which pathogens or allergens could modify their production.

The study by Jiao and Sun summarizes new advances in the understanding of bacterial manipulation of autophagic responses. The authors investigate the multiple levels at which microbial effectors interfere with this pathway to prevent pathogen recognition by the host. Improving the knowledge of these interactions will be a prerequisite for the development of more effective, specific therapies against several chronic pathologies.

One of the most frequent triggers of chronic inflammation is the disruption of the epithelial barrier. In line of evidence, Wei et al. demonstrate that the hybrid peptide cecropin-LL37 can prevent intestinal infection caused by enterohemorrhagic *Escherichia coli* by promoting an anti-inflammatory response, which reduced enterocyte apoptosis more effectively than previously developed compounds. In a similar perspective, Luo X. et al. propose a model of dextran sulfate sodium (DSS)-induced inflammatory bowel disease to evaluate the effects of obacunome, a natural citrus-derived compound. They found a marked suppression of the severe inflammatory response mediated through interference with toll-like receptor 4 (TLR4)/nuclear factor-kappa B (NF-κB) signaling. Furthermore, obacunone administration resulted in increased expression of proteins associated with the enterocytes' tight junctions and recovery of epithelial homeostasis.

The elegant work by Jang et al. investigates the mechanisms underlying IL-1β production by neutrophils in response to *Helicobacter pylori* infection. These gram-negative bacteria, which cause gastrointestinal diseases in humans, triggers massive production of IL-1β considered a key factor in inducing gastrointestinal malignancies. The authors report that neutrophils require NLRP3, ASC and caspase to secrete IL-1β in response to *H. pylori* suggesting an essential role for the NLRP3 inflammasome. TLR2, but not TLR4 and Nod2, also contributed to IL-1β secretion. On the other hand, *H. pylori* depends on both the bacterial-mediated type IV secretory system (T4SS) and motility to induce IL-1β production by neutrophils.

Schulz-Kuhnt et al. addresses the role of CCR6^+^ILC2 cells in patients with cystic fibrosis (CF). Having observed a substantial decrease in this population in patients' blood, the authors hypothesized that this might reflect pulmonary recruitment. To test this assumption, they used a new, sophisticated humanized mouse model with light-sheet fluorescence microscopic visualization, which enabled them to assess whether human ILC2 cells had accumulated in the lung following papain-induced airway inflammation. Upon adoptive transfer, CCR6^+^ILC2 cells from healthy donors did effectively infiltrate the lung of these mice, enhanced local eosinophil and neutrophil accumulation and up-regulated type-VI collagen expression. These findings support the notion that in CF patients CCR6^+^ILC2 cells might contribute to pulmonary damage once they have entered this tissue. Further analyses in lung biopsies of CF patients are required to confirm or infirm this assumption.

In their contribution Kindermann et al. likewise address the question of the involvement ILC2 cells in pulmonary infections using a model based on *Cryptococcus neoformans* injections. The authors observed an important increase in ILC2 cell counts in the lung of infected mice, accompanied by the induction of a type-2 immune response. Conversely, ILC2-deficient mice displayed increased type-1 immunity, classical macrophage activation, reduced lung inflammation and prolonged survival. These observations suggest that ILC2 cells could act as negative regulators of protective, type-1 anti-cryptococci immune responses.

Alberca-Custodio et al. propose a novel immunotherapy strategy against allergens, based on a liposomal formulation that contains the allergen at low doses associated with a TLR9 antagonist. In allergic asthma models using ovalbumine or mite extracts, the liposomal formulation containing co-encapsulated allergen plus CpG-ODN reversed an established allergic lung inflammation and provided long-term protection. It showed efficacy in reducing the inflammatory response by interacting with CD11c^+^ cells via MyD88 signaling.

In allergies, the central role of mast cells as initiators of the symptoms associated with hypersensitivity reactions is widely accepted. However, little is known about the implication of connective tissue mast cells (MMCs) in allergic inflammatory responses. Lou Y. et al. offer researchers in this field a novel means of investigation, having developed genetically modified mice lacking this particular type of mast cells. Their model, obtained by crossing α-chymase-Cre transgenic with a strain bearing a floxed allele of the myeloid cell leukemia sequence 1 (Mcl-1), provides an innovative tool to learn more about the role of mast cells, especially MMCs, in the development of allergy and will likely contribute to the development of new therapeutic strategies.

Finally, the review of Sui and Berzofsky, focuses on the new concept of trained innate immunity. The authors discuss the “memory and specificity” of innate immune populations, such as myeloid and NK cells including the contribution of epigenetics, metabolism as well as a variety of agents, such as adjuvants, in the developments of trained immunity. They focus more particularly on trained innate immunity induced by vectors/adjuvants that have been used in AIDS vaccine studies and propose new ways of thinking about these additives.

The contributions to this special topic highlight recent advances in the understanding of the complex mechanisms leading to tissue damage following activation of the innate effectors and/or their mediators. They lend further support to the importance of this first line of defense to safeguard mucosal homeostasis, which has been increasingly consolidated during the last years. By identifying new therapeutic targets they pave the way for the development of specific strategies for the treatment of chronic pathologies affecting mucosal tissues.

## Author Contributions

L-MM, CM, and VM edited the topic and wrote the manuscript. All authors contributed to the article and approved the submitted version.

## Conflict of Interest

The authors declare that the research was conducted in the absence of any commercial or financial relationships that could be construed as a potential conflict of interest.

